# Transcatheter pulmonary valve replacement with the venusP-valve system in a patient with double outlet right ventricle and transposition of the great arteries a first-in-human case report

**DOI:** 10.3389/fcvm.2025.1672008

**Published:** 2025-12-05

**Authors:** Federica Gramegna, Paolo Denti, Matteo Saccocci, Nicola Buzzatti, Alessandro Faggi, Eustachio Agricola, Ottavio Alfieri, Massimo Chessa, Francesco Maisano

**Affiliations:** 1 Department of Cardiac Surgery, Heart Valve Center, IRCCS San Raffaele Hospital, Milan, Italy; 2 Departement of Cardiovascular Imaging, IRCCS San Raffaele Hospital, Milan, Italy; 3 UniSR -Vita Salute San Raffaele University, Milan, Italy; 4 ACHD Unit—Pediatric and Adult Congenital Heart Centre, IRCCS-Policlinico San Donato, Milan, Italy

**Keywords:** pulmonary valve, venusP valve, double outlet right ventricle, transposition of the great arteries, transcatheter valve implantation

## Abstract

The Taussig-Bing anomaly is a rare form of double outlet right ventricle (DORV) characterized by complex physiology, often requiring multiple surgeries. This report describes the use of the VenusP-Valve System ina 36-year-old patient with Taussig-Bing anomaly, transposition of the great arteries (TGA), and prior surgical interventions including Blalock–Taussig shunt, arterial switch, and ventricular septal defect (VSD) closure. The patient presented with atrial fibrillation, baseline clinical signs of right-sided heart failure (jugular venous distension, edema, holosystolic murmur), New York Heart Association (NYHA) III symptoms, severe tricuspid regurgitation (TR), residual VSD, and significant pulmonary stenosis. Right-heart catheterization revealed an extremely high baseline Qp/Qs of 10.4, derived by the Fick method with oxygen consumption estimation and mixed venous sampling; this value was interpreted as artifact-influenced, and normalized to ∼2 after medical optimization. After multidisciplinary discussion, a transcatheter pulmonary valve replacement (TPVR) using the VenusP-Valve System was successfully performed. At 30 days, the patient improved to NYHA II with no major complications, reduction of TR to moderate, and stable residual VSD shunt (Qp/Qs ∼2). This case highlights the feasibility and clinical utility of VenusP-Valve in managing complex congenital heart disease, particularly in enlarged, irregular, patch-augmented right ventricular outflow tracts (RVOTs) not suitable for balloon-expandable or hourglass-anchoring valves.

## Introduction

The Taussig-Bing anomaly, a variant of DORV, involves both great arteries originating from the right ventricle with a subpulmonary VSD (1). Surgical correction typically includes arterial switch and VSD closure, but patients may require multiple interventions (2). Post-surgical RVOT anatomy is often enlarged, irregular, or patch-augmented, posing challenges for subsequent valve replacement. This report presents a transcatheter approach using the VenusP-Valve System in a patient with TGA and DORV.

## Case presentation

**Figure 1:**
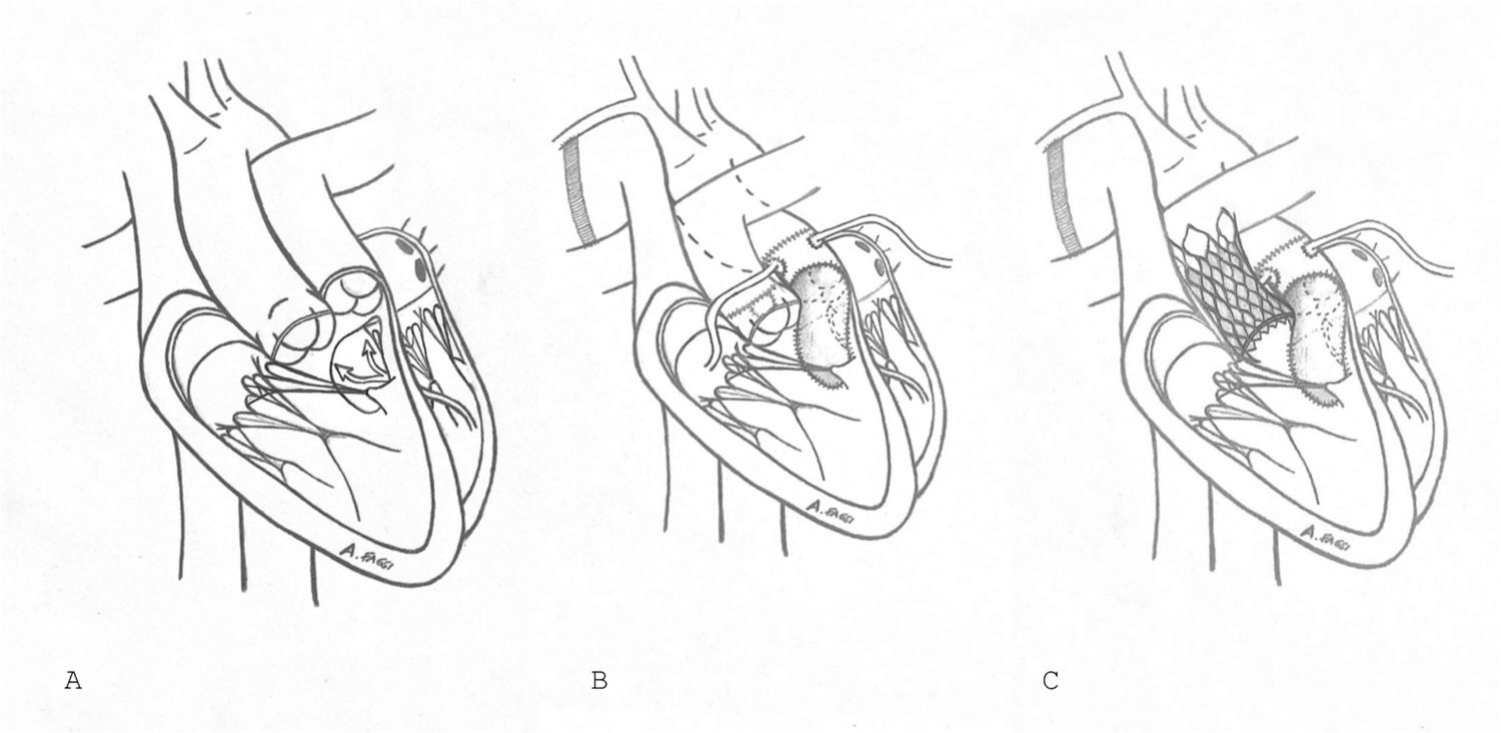
Visual summary. **(A)** Double outlet right ventricle with trasposition of great vessels. **(B)** Correction of great vessels transposition after Blalock–Taussig shunt. **(C)** Impantation of VenusP valve into pulmonary valve.

A 36-year-old male with known Taussig-Bing anomaly underwent initial palliation with a Blalock–Taussig shunt, followed by arterial switch and attempted VSD closure (Figure 1). He presented with chronic atrial fibrillation, jugular venous distension, peripheral edema, resting O₂ saturation of 92%, and NYHA class III dyspnea. CT and transoesophageal echocardiography (TOE) revealed severe TR, residual VSD, thickened neo-pulmonary trunk, and pulmonary stenosis. CT confirmed an enlarged, irregular, patch-augmented RVOT, favoring VenusP sizing. Precise numeric diameters were not archived, which is acknowledged as a limitation.

Right-heart catheterization revealed Qp/Qs 10.4 by the Fick method, with artifact-related overestimation; after diuretic optimization, Qp/Qs normalized to ∼2.0. Trans-RVOT gradient and RV systolic pressures were available, whereas complete invasive pressures and oxygen saturations could not be retrieved retrospectively and are reported as a limitation.

TPVR was performed via right femoral vein using a 34 mm VenusP-Valve. A 26F sheath was advanced, a stiff wire and rail were positioned, anticoagulation maintained ACT >250 s, and the valve was deployed under fluoroscopic and echocardiographic guidance. No rapid pacing was used. Contrast and radiation doses were not archived. Valve positioning was verified with angiography and echocardiography. Deployment was successful without complications.

At 30 days, echocardiography showed no pulmonary stenosis, trivial pulmonary regurgitation, TR reduced to moderate, residual VSD shunt stable with Qp/Qs ∼2, and reduced RV systolic pressure. The patient improved to NYHA II, with relief of dyspnea and no arrhythmias.

## Discussion

This case illustrates the complexity of managing congenital patients after arterial switch, where factors such as altered RVOT geometry, scarring from multiple surgeries, and coronary reimplantation must be considered. In this setting, choosing the appropriate valve platform is critical.

Comparative valve features are summarized in Table 1. Balloon-expandable valves such as Sapien 3 offer high radial force but require rigid prestented conduits (3–5). The Harmony valve, although self-expanding, anchors via an hourglass design and is suitable mainly for smoothly dilated, compliant RVOTs (6). The Melody valve, a balloon-expandable valve, is limited to narrow conduits and requires prestenting (7). In contrast, the VenusP-Valve provides strong radial force with dual flares, allowing anchoring in enlarged or irregular RVOTs without prestenting (8, 9).

**Table 1:** Comparative features of available transcatheter pulmonary valve platforms.

Valve	Type	Radial force	Anchoring mechanism	Anatomical requirements	Prestenting	Regulatory status
VenusP-Valve	Self-expanding nitinol	High	Dual proximal & distal flares	Enlarged, irregular or patch-augmented RVOTs	Not required	CE-marked (Europe), under evaluation in US
Harmony Valve	Self-expanding nitinol	Moderate	Hourglass design with inflow/outflow flares	Compliant, smoothly dilated RVOTs	Not required	FDA-approved (US), CE-marked (Europe)
Sapien 3	Balloon-expandable cobalt-chromium	High	Radial force against rigid prestented conduit	Requires rigid prestented or conduit landing zone	Required	FDA & CE-marked
Melody Valve	Balloon-expandable platinum–iridium	Moderate	Radial force in prestented conduit or homograft	Narrow conduits/homografts, limited size range	Required	FDA & CE-marked

RVOT, right ventricular outflow tract; FDA, Food and Drug Administration; CE, Conformité Européenne.

Mechanistically, VenusP expands against the thickened RVOT, relieving obstruction and restoring laminar flow. Afterload reduction and improved RV geometry contributed to the observed decrease in TR. Residual VSD remained stable, supporting a staged management approach, as described in adult congenital practice (10).

Although not the first application of transcatheter pulmonary valves in this anatomy, this case adds to evidence supporting VenusP-Valve feasibility in complex congenital heart disease. It underscores the importance of individualized planning based on anatomy, valve characteristics, and surgical history.

## Patient perspective

The patient reported significant improvement in symptoms and quality of life post-procedure.

## Conclusion

VenusP-Valve TPVR is a feasible and effective option in high-risk congenital heart disease cases. Its deployment avoids high-risk re-sternotomy and supports continued expansion of percutaneous options in complex anatomies.

## Data Availability

The raw data supporting the conclusions of this article will be made available by the authors, without undue reservation.
